# Expanding severe acute respiratory infection (SARI) surveillance beyond influenza: The process and data from 1 year of implementation in Vietnam

**DOI:** 10.1111/irv.12571

**Published:** 2018-06-10

**Authors:** Karen A. Alroy, Trang Thuy Do, Phu Dac Tran, Tan Quang Dang, Long Ngoc Vu, Nga Thi Hang Le, Anh Duc Dang, Nghia Duy Ngu, Tu Huy Ngo, Phuong Vu Mai Hoang, Lan Trong Phan, Thuong Vu Nguyen, Long Thanh Nguyen, Thinh Viet Nguyen, Mai Quang Vien, Huy Xuan Le, Anh The Dao, Trieu Bao Nguyen, Duoc Tho Pham, Van Thi Tuyet Nguyen, Thanh Ngoc Pham, Binh Hai Phan, Brett Whitaker, Thuy Thi Thu Do, Phuong Anh Dao, S. Arunmozhi Balajee, Anthony W. Mounts

**Affiliations:** ^1^ Division of Viral Diseases National Center for Immunization and Respiratory Diseases Centers for Disease Control and Prevention Atlanta GA USA; ^2^ Division of Global Health Protection Center for Global Health Centers for Disease Control and Prevention Hanoi Vietnam; ^3^ General Department of Preventive Medicine Ministry of Health Hanoi Vietnam; ^4^ National Institute of Hygiene and Epidemiology Hanoi Vietnam; ^5^ Pasteur Institute in Ho Chi Minh City Ho Chi Minh City Vietnam; ^6^ Pasteur Institute in Nha Trang Khanh Hoa Vietnam; ^7^ Tay Nguyen Institute of Hygiene and Epidemiology Dak Lak Vietnam; ^8^ Division of Global Health Protection Center for Global Health Centers for Disease Control and Prevention Atlanta GA USA

**Keywords:** adenovirus, global health security, influenza virus, respiratory syncytial virus, rhinovirus, severe acute respiratory infection, Vietnam

## Abstract

**Background:**

In 2016, as a component of the Global Health Security Agenda, the Vietnam Ministry of Health expanded its existing influenza sentinel surveillance for severe acute respiratory infections (SARI) to include testing for 7 additional viral respiratory pathogens. This article describes the steps taken to implement expanded SARI surveillance in Vietnam and reports data from 1 year of expanded surveillance.

**Methods:**

The process of expanding the suite of pathogens for routine testing by real‐time reverse transcriptase–polymerase chain reaction (rRT‐PCR) included laboratory trainings, procurement/distribution of reagents, and strengthening and aligning SARI surveillance epidemiology practices at sentinel sites and regional institutes (RI).

**Results:**

Surveillance data showed that of 4003 specimens tested by the RI laboratories, 20.2% (n = 810) were positive for influenza virus. Of the 3193 influenza‐negative specimens, 41.8% (n = 1337) were positive for at least 1 non‐influenza respiratory virus, of which 16.2% (n = 518), 13.4% (n = 428), and 9.6% (n = 308) tested positive for respiratory syncytial virus, rhinovirus, and adenovirus, respectively.

**Conclusions:**

The Government of Vietnam has demonstrated that expanding respiratory viral surveillance by strengthening and building upon an influenza platform is feasible, efficient, and practical.

## 1 INTRODUCTION

In support of the World Health Organization (WHO) and its global network of National Influenza Centers (NIC), the U.S. Centers for Disease Control and Prevention (CDC) has provided pivotal technical assistance to more than 50 countries in the development of surveillance and laboratory capacity for influenza virus.^1^, ^2^ These efforts have helped nations describe influenza seasonality, identify influenza isolates for vaccines, and develop plans for pandemic preparedness. Regional and international initiatives such as the Asia Pacific Strategy for Emerging Diseases (APSED)^3^ and the Global Health Security Agenda (GHSA)^4^ have further strengthened the ability of many countries to comply with the International Health Regulations (IHR), a 2005 legal mandate among all WHO member states to protect global public health security.

Many countries that conduct surveillance for severe acute respiratory infection (SARI) routinely test SARI specimens only for the presence of influenza virus^5^ although other respiratory viruses clearly play an important role in causing severe respiratory infections.^6^ Globally, influenza is identified as the etiologic agent in only about 10%‐20% of SARI surveillance cases.^7^, ^8^, ^9^ Without adequate reagents and routine procedures for testing non‐influenza respiratory viruses,^10^ the pathogens causing the remaining 80%‐90% of cases from SARI sentinel surveillance are not identified. Data on non‐influenza respiratory viruses are essential for public health institutions to understand the burden of disease due to these pathogens.

In Vietnam, at the national level, the Ministry of Health General Department of Preventive Medicine (GDPM) is responsible for the country’s surveillance efforts. In 2016, GDPM embarked on strengthening and expanding SARI surveillance with technical support from WHO and CDC, funded through the GHSA. The following improvements were instituted in Vietnam’s SARI surveillance program: (i) revision of the laboratory testing algorithm and provision of select, standardized laboratory reagents for detection of non‐influenza respiratory viruses; (ii) training for non‐influenza respiratory viral diagnostics; (iii) establishment of a proficiency testing program for these viruses; and (iv) harmonization of epidemiologic approaches leading to the development of a national SARI surveillance protocol including standardized case definitions, detailed instructions for safe specimen collection and transport, and standard operating procedures for testing and reporting results. This study describes the expansion and experience in detail.

## 2 METHODS

### 2.1 Laboratory algorithm and reagent procurement

**Figure 1:**
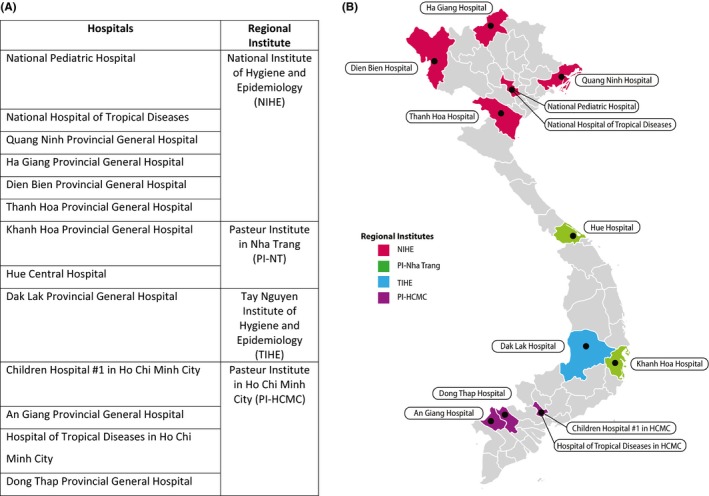
A, List of the SARI sentinel sites selected at the harmonization workshop. B, Map illustrating SARI sentinel sites’ geographic distribution across Vietnam and the Regional Institute that cooperates with each sentinel site

In 2005, Vietnam established a program for outpatient surveillance of influenza‐like illness (ILI) and, in 2011, added hospital‐based sentinel surveillance of SARI. Vietnam’s 4 regional institutes (RI) have each independently managed the sentinel sites and laboratory testing for their respective region. The National Institute of Hygiene and Epidemiology (NIHE), the Pasteur Institute in Ho Chi Minh City (PI‐HCMC), the Pasteur Institute in Nha Trang (PI‐NT), and the Tay Nguyen Institute of Hygiene and Epidemiology (TIHE) cover the north, south, central coast, and central highlands regions, respectively (Figure 1). SARI surveillance activities in Vietnam have been approved as routine public health surveillance activities and therefore do not involve human subject research, by the human subjects research office in the CDC’s Center for Global Health (CGH #2016‐201).

**Figure 2:**
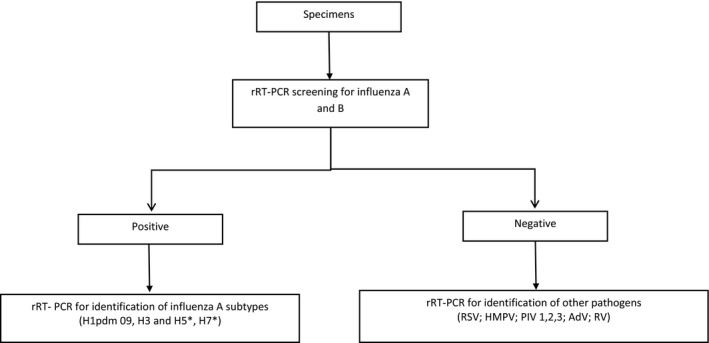
Testing algorithm for expanded SARI surveillance in Vietnam. *If samples are positive with subtypes of H5 and H7N9, the labs at TIHE and PI‐Nha Trang will send samples to PI‐ HCMC and NIHE, respectively, for confirmation

Until 2016, all SARI surveillance samples in Vietnam were tested for influenza A and influenza B, and positive samples underwent additional subtyping. Through this study, this algorithm was modified with the following change: all specimens that tested negative for influenza were tested for each of 7 non‐influenza viral pathogens—respiratory syncytial virus (RSV); human metapneumovirus (hMPV); human parainfluenza viruses 1 (PIV1), 2 (PIV2), and 3 (PIV3); human rhinovirus (RV); and human adenovirus (AdV)—in a series of 7 singleplex reactions (Figure 2). Following this modification, the SARI surveillance platform was referred to as expanded SARI surveillance to reflect the additional pathogens that were included in routine testing.

The International Reagent Resource (IRR, formerly known as the Influenza Reagent Resource, https://www.internationalreagentresource.org/), an existing online reagent portal managed by the Influenza Division at CDC, was modified to include primers and probes for these 7 non‐influenza respiratory viruses in order to ensure availability of quality reagents. Vietnam’s NIC laboratories, NIHE and PI‐HCMC, already had access to the IRR for influenza surveillance; through this current study, TIHE and PI‐NT were newly authorized to receive these reagents. All reagents procured through the IRR were available to laboratories at no cost.

### 2.2 Training for non‐influenza viral diagnostics and proficiency testing

To ensure accurate use of these diagnostic tests, CDC engaged the RIs in a series of trainings. This included a 4‐day in‐country training of didactic and laboratory instruction held at the PI‐HCMC in August 2015. Topics covered laboratory safety; setup of a molecular diagnostics laboratory; appropriate specimen collection, transport, and storage; overview of real‐time reverse transcriptase–polymerase chain reaction (rRT‐PCR); software data analysis of rRT‐PCR; and a discussion on the implementation of expanded SARI surveillance. Regional institute personnel were trained to test for the panel of 7 non‐influenza respiratory viruses as well as to test for Middle East Respiratory Syndrome coronavirus (MERS‐CoV) to increase preparedness in the event of an outbreak.^11^, ^12^, ^13^, ^14^, ^15^, ^16^ Following this training, each RI had an additional site visit by CDC experts, who offered guidance that was specific to the needs of each laboratory.

After the first training, each of the RIs was provided with an external quality assessment (EQA) panel for respiratory viruses. This EQA panel provided the laboratory with a series of samples intended to mimic human respiratory specimens. While the types of pathogen were known by CDC, this information was not shared with the laboratories in an attempt to test the accuracy and proficiency of each laboratory’s rRT‐PCR capabilities. The RIs received an EQA with 2 components, non‐influenza respiratory viruses and MERS‐CoV. The non‐influenza respiratory portion had a 7‐component panel that was available commercially (http://www.qcmd.org). This panel contained RSV, hMPV, RV, AdV, and PIV1‐3. The MERS‐CoV EQA panel was manufactured by CDC and contained 5 mock specimens spiked with inactivated MERS‐CoV.

The EQA specimens were processed according to the CDC non‐influenza respiratory virus rRT‐PCR method, as part of the expanded SARI surveillance protocol. The results were submitted to CDC for evaluation and each laboratory received a confidential individual performance report. Expected EQA performance was determined by compiling baseline data at CDC using the same panels, assays, and protocols that were used in Vietnam.

A second 4‐day training took place in Atlanta, Georgia, in July 2016, hosted by CDC and the Association of Public Health Laboratories (APHL). This included didactic and laboratory work, and was held at the Laboratory of the Georgia Department of Public Health. This curriculum reinforced prior topics as well as introduced new ones including quality assurance/quality control, software for data analyses, and information on reagent acquisition through the IRR.

### 2.3 Harmonization and expansion of SARI surveillance

Vietnam launched its expanded SARI surveillance initiative by holding a harmonization workshop in March 2016. The workshop was hosted by GDPM and attended by personnel from the 4 RIs, CDC, WHO, USAID, and PATH, formerly the Program for Appropriate Technology and Health, an international non‐governmental organization that has supported the development of a web‐based platform for SARI surveillance reporting. The 2‐day workshop aimed to align surveillance practices and ensure that all parties shared a common vision of the new expanded surveillance process. Key elements identified at the workshop for strengthening and improvement included case detection, specimen transport and storage, and laboratory testing. Following the workshop, these elements were reviewed and revised in the existing SARI surveillance guidance. Before 2016, several of the sentinel sites and RIs were operating SARI surveillance differently in terms of case definitions, testing algorithms, and epidemiology data collection forms. Not all sentinel sites had adopted the 2014 revised SARI case definition, recommended internationally by WHO. At the harmonization workshop, all sentinel sites agreed to use the WHO 2014 SARI case definition.

Patient enrollment was discussed and aligned during the harmonization workshop as follows: The first 2 cases of the day that met the SARI case definition were selected for SARI enrollment to minimize selection bias. Patients of all ages from the participating wards would be considered for patient selection as long as they met the SARI case definition. As was previously done for SARI sentinel influenza surveillance, data collection forms were completed via patient interview and nasopharyngeal and oropharyngeal swabs were collected from each enrolled patient. In severe cases, endotracheal aspirates were also accepted as specimen types.

At the harmonization workshop, unified, practical specimen transport guidance was agreed upon to maintain sample integrity and promote sustainability. According to this guidance, both nasopharyngeal and oropharyngeal swabs from an individual patient were stored in a single viral transport media (VTM) tube and transported once a week to the RIs by routine transportation systems. Previously, the specimens were transported as soon as specimens were collected, resulting in an unsustainable and expensive transportation process.

At the harmonization meeting, sentinel sites for expanded SARI surveillance were selected. The following criteria were used for selecting the sites: prior engagement and/or performance with SARI surveillance, representation from Vietnam’s 8 ecological regions, and commitment from the sentinel sites. A total of 13 sentinel sites, 9 supported by CDC and 4 supported by WHO, agreed to conduct expanded SARI surveillance, cooperate fully with the RIs, and strictly adhere to the protocols outlined in the revised SARI Surveillance Guidelines (Figure 1). Following the harmonization workshop, the RIs reinforced these concepts to the sentinel sites through periodic monitoring visits enabling guidance and support.

The data reported in this article are from specimens collected and tested following the revised SARI Surveillance Guidelines.

### 2.4 Laboratory testing and data reporting

Specimens were collected at the sentinel sites and delivered once a week to the laboratories of the RIs. Each of the RIs utilized the reagents procured through the IRR for specimen analysis. At the RI laboratories, total nucleic acid (TNA, ie, both ribonucleic acids and deoxyribonucleic acids) was extracted from the specimens. The 7 non‐influenza pathogens were tested by singleplex rRT‐PCR assays, designed by the Division of Viral Diseases at the National Center for Immunization and Respiratory Diseases, CDC.^11^, ^12^, ^13^, ^14^, ^15^, ^16^

Each week, epidemiology and laboratory data from the 4 RIs were sent to the epidemiology team at NIHE who prepared a weekly report, which was then distributed electronically to key public health stakeholders and partners. Two notable modifications were implemented to strengthen the data reporting process. GDPM designed improved SARI surveillance data reporting forms for RIs to reduce manual data cleaning at the regional and national levels, and GDPM, CDC, and PATH worked collaboratively with the RIs to integrate the SARI surveillance data into the digitized electronic data warehouse managed by the public health emergency operation center at GDPM.^17^

## 3 RESULTS

From January 2016 until May 2017, there were 13 sentinel sites and 4 RIs that participated in expanded SARI surveillance (Table 1). The following results explain the SARI surveillance expansion in Vietnam.

**Table 1:**
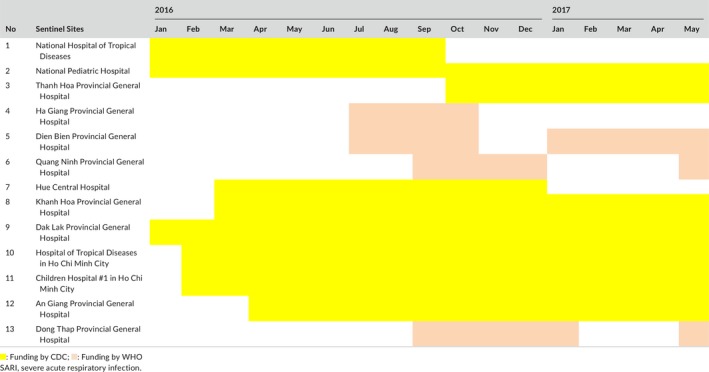
Timeline illustrating sentinel sites’ funding and collection of data for expanded SARI surveillance, and the organization supporting these sites

### 3.1 Laboratory algorithm and reagent procurement

The RIs implemented the modified testing algorithm that was agreed during the harmonization meeting in March 2016. This new laboratory algorithm for testing SARI specimens was emphasized during the in‐country and the US‐based laboratory trainings.

The RIs were provided with an initial reagent inventory to start, comprised of 1 primer and probe kit for the Non‐Influenza Respiratory Virus rRT‐PCR Panel (IRR product #GR‐125) and 1 Non‐Influenza Respiratory Virus rRT‐PCR Positive Control (IRR product #GR‐77). This quantity of reagent was sufficient for each laboratory to test approximately 800‐900 specimens. Together, the RI laboratories made 8 subsequent orders from the IRR for expanded SARI surveillance reagents between September 2016 until July 2017.

### 3.2 Training for non‐influenza viral diagnostics and proficiency testing

Each RI sent 3‐4 individuals to participate in the 4‐day training at PI‐HCMC. Two Vietnamese laboratorians, 1 from NIHE and 1 from PI‐HCMC, participated in the training workshop in Atlanta.

The 4 RIs completed the proficiency test panels, and their results were shared electronically with CDC. These results were evaluated for correct pathogen identification among a range of specimens. All 4 laboratories passed the EQA panels with 100% correct results.

### 3.3 Harmonization and expansion of SARI surveillance

After the harmonization workshop, the SARI Surveillance Guidelines were revised and the sentinel sites agreed to participate in expanded SARI surveillance using the new protocol. The SARI Surveillance Guidelines were approved by the Ministry of Health in April 2017, making the document official regulation for national SARI surveillance. Sentinel sites began implementing the expanded SARI surveillance as early as January 2016; however, the majority of sites were collecting data by March 2016 (Table 1). One hospital withdrew their participation, reducing the total number of active sites to 12. NIHE and GDPM discontinued 1 SARI site, the National Hospital of Tropical Diseases, in October 2016 and added Thanh Hoa Provincial General Hospital to improve geographic distribution in Northern Vietnam and representation of the patient population. Ha Giang Provincial General Hospital stopped participating in SARI surveillance in November 2016. Hue Central Hospital temporarily stopped collecting SARI samples from January 2017 until the time of writing due to administrative barriers. Other interruptions to data collection in Dien Bien, Quang Ninh, and Dong Thap sentinel sites resulted from delays in funding reaching the sites. Within the calendar year of 2016, the 4 RIs conducted a total of 42 monitoring visits to their respective sentinel sites, ranging between 2 and 4 monitoring visits per site per year.

### 3.4 Laboratory analysis and data reporting

Between March 2016 and March 2017, a total of 41 271 patients met the SARI case definition at the sentinel sites. Specimens from 4003 SARI patients were collected and sent to the RI laboratories for testing. The age distribution of sampled SARI patients and the median age per site are reported in Table 2. A total of 810 (20.2%) specimens were positive for influenza virus, with the following breakdown: 307 influenza A H1N1, 216 influenza A H3N2, and 298 influenza B (Table 3). Eleven specimens were positive for 2 influenza viruses.

**Table 2:** The total number of patients admitted to the sentinel hospitals that met the SARI case definition, the number of those SARI patients from whom specimens were taken, and the age distribution and median age of sampled SARI patients from each sentinel site

Sentinel sites	Total number of SARI patients	Number of SARI patients tested	Age of SARI patients tested			
			<1 y	1 to <5 y	≥5 y	Median age of SARI patients tested
National Pediatric Hospital (NPH)	10 142	506	293 (55.7%)	177 (35.0%)	36 (7.1%)	0.8
National Hospital of Tropical Diseases (NHTD)	258	113	2 (1.8%)	1 (0.9%)	110 (97.3%)	45.5
Ha Giang Provincial General Hospital	211	160	0	7 (4.4%)	153 (95.6%)	29.5
Dien Bien Provincial General Hospital	318	165	1 (0.6%)	14 (8.5%)	150 (90.9%)	34
Quang Ninh Provincial General Hospital	201	80	0	0	80 (100%)	52
Thanh Hoa Provincial General Hospital	217	74	0	0	74 (100%)	54
Hospital of Tropical Diseases in HCMC	443	398	13 (3.3%)	110 (27.6%)	275 (69.1%)	39.9
Children Hospital #1 in HCMC	17 071	505	136 (26.9%)	343 (67.9%)	26 (5.1%)	3.1
An Giang Provincial General Hospital	1113	426	0	0	426 (100%)	53.4
Dong Thap Provincial General Hospital	1892	180	84 (46.7%)	58 (32.2%)	38 (21.1%)	14.3
Khanh Hoa Provincial General Hospital	3073	470	128 (27.2%)	83 (17.7%)	259 (55.1%)	2.2
Hue Central Hospital	3343	416	194 (46.6%)	207 (49.8%)	15 (3.6%)	1
Dak Lak Provincial General Hospital	2989	510	103 (20.2%)	134 (26.2%)	273 (53.5%)	17
Total	41 271	4003	954 (23.8%)	1134 (28.3%)	1915 (47.8%)	

SARI, severe acute respiratory infection.

**Table 3:** Results of expanded SARI surveillance testing in Vietnam by sentinel site, provides data on influenza viruses, hMPV, AdV, PIV1‐3, RSV, and RV

	Influenza viruses									Non‐Influenza Respiratory Viruses														
Sentinel sites	No. SARI tested	No. samples pos for influenza		A/H1N1/09		A/H3N2		B		# samples neg for influenza	hMPV		AdV		PIV1		PIV2		PIV3		RSV		RV	
	No.	No.	%	No.	%	No.	%	No.	%	No.	No.	%	No.	%	No.	%	No.	%	No.	%	No.	%	No.	%
National Pediatrics Hospital (NPH)	506	88	17.4	47	9.3	21	4.2	23	4.5	418	21	5.0	57	13.6	22	5.3	3	0.7	37	8.9	129	30.9	80	19.1
National Hospital of Tropical Diseases	113	36	31.9	17	15.0	8	7.1	13	11.5	77	1	1.3	0	0.0	0	0.0	0	0.0	1	1.3	0	0.0	2	2.6
Ha Giang Provincial General Hospital	160	24	15.0	8	5.0	15	9.4	1	0.6	136	0	0.0	7	5.1	3	2.2	2	1.5	5	3.7	3	2.2	3	2.2
Dien Bien Provincial General Hospital	165	12	7.3	0	0.0	7	4.2	5	3.0	153	2	1.3	6	3.9	3	2.0	1	0.7	0	0.0	8	5.2	9	5.9
Quang Ninh Provincial General Hospital	80	5	6.3	0	0.0	5	6.3	0	0.0	75	0	0.0	1	1.3	3	4.0	0	0.0	2	2.7	0	0.0	1	1.3
Thanh Hoa Provincial General Hospital	74	12	16.2	3	4.1	5	6.8	4	5.4	62	1	1.6	2	3.2	3	4.8	0	0.0	0	0.0	3	4.8	3	4.8
Hospital of Tropical Disease in Ho Chi Minh City	398	112	28.2	62	15.6	28	7.0	22	5.5	286	12	4.2	17	5.9	4	1.4	0	0.0	11	3.8	8	2.8	25	8.7
Children Hospital # 1 in Ho Chi Minh City	505	80	15.9	38	7.5	10	2.0	32	6.3	425	29	6.8	24	5.6	17	4.0	9	2.1	26	6.1	85	20.0	64	15.1
An Giang Provincial General Hospital	426	82	19.3	29	6.8	23	5.4	30	7.0	344	10	2.9	1	0.3	2	0.6	0	0.0	0	0.0	7	2.0	25	7.3
Dong Thap Provincial General Hospital	180	18	10.0	6	3.3	3	1.7	9	5.0	162	29	14.1	23	3.7	5	0.6	0	0.0	16	3.1	49	20.3	45	14.7
Khanh Hoa Provincial General Hospital	470	137	29.1	40	8.5	38	8.1	61	13.0	333	25	7.5	52	15.6	19	5.7	1	0.3	27	8.1	70	21.0	50	15.0
Hue Central Hospital	416	74	17.8	19	4.6	22	5.3	36	8.7	342	18	5.3	89	26.0	23	6.7	4	1.2	33	9.6	114	33.3	90	26.3
Dak Lak Provincial General Hospital	510	130	25.5	38	7.5	31	6.1	62	12.2	380	8	2.1	29	7.6	11	2.9	9	2.4	17	4.5	42	11.1	31	8.2
**Total**	4003	810	20.2	307	7.7	216	5.4	298	7.4	3193	156	4.9	308	9.6	115	3.6	29	0.9	175	5.5	518	*16.2*	428	13.4

SARI, severe acute respiratory infection; hMPV, human metapneumovirus; AdV, adenovirus; PIV1‐3, parainfluenza 1,2,3; RSV, respiratory syncytial virus; RV, rhinovirus.

All 3193 influenza‐negative samples were tested for each of the 7 non‐influenza respiratory viruses. Of these, 1337 (41.8%) specimens were positive for at least 1 non‐influenza respiratory virus. The number and proportion positive for each virus is as follows: 518 (16.2%) RSV, 428 (13.4%) RV, 308 (9.6%) AdV, 175 (5.5%) PIV3, 156 (4.9%) hMPV, 115 (3.6%) PIV1, and 29 (0.9%) PIV2 (see Table 3 for results by sentinel site). Nearly half of all SARI specimens—1856 (46.4%)—were negative for influenza virus as well as the 7 common non‐influenza respiratory viruses. Of all of the samples tested for non‐influenza viruses, 11% were positive for more than 1 virus. There were 314 specimens with 2 viruses, 36 with 3 viruses, and 2 specimens with 4 viruses. Combinations of AdV, RSV, and RV constituted the 3 most common co‐infection combinations. Table 4 provides a detailed breakdown of these data.

**Table 4:** Combination and number of non‐influenza respiratory viral co‐infections from expanded SARI

Virus combination	# SARI cases	Virus combination	# SARI cases
AdV – RV	63	PIV3 – RSV – RV	3
RSV – RV	52	hMPV – AdV – PIV3	3
AdV – RSV	48	PIV2 – RV	2
PIV3 – RV	27	PIV1 – RSV – RV	2
PIV3 – RSV	22	PIV1 – PIV3 – RSV	2
AdV – PIV3	21	AdV – PIV3 – RSV	2
hMPV – RV	15	AdV – PIV1 – RSV	2
hMPV – AdV	12	AdV – PIV1 – PIV3	2
PIV1 – RV	10	PIV2 – RSV	1
PIV1 – RSV	10	PIV2 – PIV3	1
AdV – PIV1	9	AdV – PIV3 – RSV – RV	1
AdV – RSV – RV	8	AdV – PIV2	1
hMPV – PIV3	8	AdV – PIV2 – RV	1
hMPV – RSV	7	hMPV – PIV3 – RV	1
AdV – PIV3 – RV	5	hMPV – PIV1	1
PIV1 – PIV3	4	hMPV – AdV – RSV	1
AdV – PIV1 – RV	4	hMPV – AdV – PIV3 – RV	1

SARI, severe acute respiratory infection.

The data generated from expanded SARI surveillance were communicated by email through routine weekly reports sharing laboratory data and presenting summary analyses. From March 2016 until March 2017, 45 weekly reports were prepared by NIHE and sent to surveillance stakeholders. The web‐based platform for SARI data entry and integration into the Vietnam data warehouse continues to be developed. In May 2017, PATH shared a demonstration version of the web‐based platform to GDPM, RIs, and sentinel sites. As of August 2017, several sentinel sites were using the platform as beta‐testers to provide feedback to PATH for improvement.

## 4 DISCUSSION

Expanding respiratory viral surveillance by strengthening and building upon an influenza platform is possible, efficient, and practical. This approach has been demonstrated by Vietnam’s Ministry of Health.

The present study shows that a relatively large proportion of SARI specimens were positive for influenza virus (20.5%). This was similar to studies in other parts of the world, including New Zealand from 2006 to 2010 (22.7%)^18^ and Thailand from 2004 to 2010 (21%).^19^ This was 4 times the proportion, however, of influenza‐positive SARI patients in Southern Vietnam between 2008 and 2010 (5.1%).^20^ While it is tempting to speculate these differences could be because of a change in disease trends, valid comparisons between these data can be challenging in light of variability in seasons and testing methods.

While influenza viruses remain a significant burden of SARI cases in Vietnam, this study showed that non‐influenza‐positive specimens were more than twice as prevalent with other respiratory viruses (41.8%). There are only few studies that have investigated non‐influenza respiratory viruses in SARI patients of Vietnam^20^ or in the South‐East Asia region.^18^, ^19^ Some studies have examined non‐influenza viruses in select patient populations such as children,^21^, ^22^, ^23^, ^24^ refugees,^25^ or hospitalized and non‐hospitalized patients with respiratory disease.^26^ Gaps still exist, however, in understanding which viruses cause SARI in Vietnam.

This study found that the most common non‐influenza respiratory viruses were respiratory syncytial virus, rhinovirus, and adenovirus (16.2%, 13.4%, and 9.6%, respectively). Research from Southern Vietnam, found that 14.3% of SARI specimens were positive for RSV.^20^ Studies of Vietnamese pediatric patients with acute respiratory infections identified 23.8% and 23% as positive for RSV.^27^, ^28^ As RSV is believed to be the most common pathogen causing acute lower respiratory infections in children,^29^ it is predictable and consistent with our findings that the proportion of RSV in pediatric‐specific populations is higher compared to SARI cases among all age categories. The proportion of rhinovirus in SARI patients found in this study (13.4%) is similar to what has been detected in hospitalized patients with respiratory symptoms in Khanh Hoa Province Vietnam in 2008‐2010 (10.2%).^26^ A study examining SARI patients from South Africa in 2009‐2010 detected that 13.3% were positive for adenovirus^30^ which is similar but slightly higher than the proportion we found in Vietnam SARI patients (9.7%). The same South African study found a much higher proportion of SARI patients positive for rhinovirus compared to our findings in Vietnam (24.9% compared to 13.4%), perhaps suggesting geographic or environmental variation.

A limitation of this study was that only influenza‐negative specimens were tested for non‐influenza respiratory pathogens. Given this testing algorithm, the study is not able to provide data on co‐infection of influenza and non‐influenza viruses. Additionally, while the presence of laboratory positive non‐influenza viruses may suggest they are an etiologic agent responsible for clinical disease, given the sensitivity of the rRT‐PCR platform and the unclear role of non‐influenza viruses in virulence, this conclusion warrants further research.^31^, ^32^, ^33^, ^34^

The 7 non‐influenza respiratory viruses used in this study were selected based on historical data demonstrating their potential involvement in respiratory infections, as well as their potential for future vaccine development.^11^, ^12^, ^13^, ^14^, ^15^, ^16^, ^35^, ^36^, ^37^, ^38^, ^39^ Of the 7 viruses, significant progress has been made toward RSV vaccine development; however, despite several potential candidates, some of which advanced to clinical trials, no vaccine so far is both safe and effective.^40^ Establishing baseline data for these viruses in people with SARI helps to provide evidence to GDPM to guide public health decision‐making. For example, if an RSV vaccine were to be developed and administered as a public health intervention in Vietnam, characterizing the benefit of the vaccine would be contingent upon knowing how severely the virus impacted the Vietnamese population before the intervention. Similarly, epidemiological data regarding these non‐influenza respiratory viruses could help GDPM prioritize how and where to focus limited public health resources.

This study provides valuable information regarding the prevalence of non‐influenza respiratory viruses in Vietnam; however, it represents only a first step toward understanding their burden of disease. While the catchment population of the sentinel sites is unknown, the representativeness of the sampled population can be considered from the makeup of the sentinel sites themselves. Eight of the sites are provincial hospitals, 4 are located in major cities, Hanoi and Ho Chi Minh City, and 2 are pediatric hospitals. In general, in Vietnam, public hospitals are visited by people with financial resources, whereas poor families often use commune health centers.^41^ In addition, healthcare utilization is reported to be 3‐4 times higher in urban areas than in the mountainous regions in the north or central regions of the country.^42^ As such, the SARI sentinel sites may be under representing poor individuals, especially from rural or remote areas. Because some of the SARI sites were pediatric hospitals, the age distribution was skewed, and 52.1% of the SARI specimens tested were in patients <5 years. This age distribution may have biased the results toward increased RSV detection. In addition, despite finding a large proportion of SARI specimens positive for RSV, it is not well understood whether the SARI case definition is appropriate for RSV,^43^ and because of this, some proportion of RSV cases may have been missed. Future research on non‐influenza respiratory viruses in Vietnam should attempt to calculate the burden of disease as well as to improve the understanding of patient clinical profiles associated with them.

In addition, while the process of expanded SARI surveillance has improved Vietnam’s ability to collect and analyze data on non‐influenza respiratory viruses, the operational costs required to sustain SARI surveillance in Vietnam are not negligible. Costs included the organization of epidemiologic and laboratory trainings, collection materials, transportation costs, laboratory reagents, human resources, and support for supervisory visits. During the expansion of the SARI surveillance platform, interruptions in funding caused delays at sentinel sites in procurement of sampling supplies; and occasionally, such interruptions halted SARI surveillance data collection at sites altogether. Delays in data reporting from the RIs in some cases hindered data aggregation and the weekly surveillance reports, although limited human resources also contributed to occasional gaps in weekly report distribution. Identifying sustainable financial support mechanisms to ensure future surveillance for SARI is currently being discussed by all parties. The Government of Vietnam is currently examining their capabilities for sustaining SARI surveillance for influenza and other respiratory viruses and considering respiratory surveillance through different event‐based surveillance approaches.

In spite of these limitations, the existence of a SARI surveillance network in Vietnam provided a platform for collecting data on important non‐influenza respiratory viruses, and the process of expanding the system offered an opportunity to revisit the data collection and analysis practices that were routine for SARI surveillance. As a multistep process, the expansion and strengthening of SARI surveillance continued to develop throughout 2016 and 2017, and will likely continue to improve with further attention and refinement to the system. The future integration of the expanded SARI surveillance data into the GDPM data warehouse offers additional potential benefit of reduced delays due to data sharing, reduced need for manual data curation, as well as provision of data to the public health emergency operation center for routine data analysis.^17^

The process of expanding SARI surveillance to test for non‐influenza respiratory viruses has engaged numerous organizations in Vietnam ranging from sentinel hospitals, RIs to GDPM and international donor organizations. Additionally, in the era of GHSA the study demonstrates an ability to enhance and expand surveillance by building and extending upon existing in‐country capacities. While these data provide valuable initial insight into the burden of severe respiratory disease, the real value will only be realized over time as data are collected and analyzed consistently through multiple years. Such data can be used to understand seasonality, contribution by various pathogens to respiratory disease burden, and the risk groups of these noninfluenza respiratory viral pathogens in Vietnam.

## DISCLAIMER

The findings and conclusions in this report are those of the authors and do not necessarily represent the official position of the Centers for Disease Control and Prevention.
